# Vertical Transmission of Zika Virus in *Aedes aegypti* Mosquitoes

**DOI:** 10.4269/ajtmh.16-0448

**Published:** 2016-11-02

**Authors:** Saravanan Thangamani, Jing Huang, Charles E. Hart, Hilda Guzman, Robert B. Tesh

**Affiliations:** 1 Department of Pathology, Institute for Human Infection and Immunity, University of Texas Medical Branch, Galveston, Texas

## Abstract

Previous experimental studies have demonstrated that a number of mosquito-borne flavivirus pathogens are vertically transmitted in their insect vectors, providing a mechanism for these arboviruses to persist during adverse climatic conditions or in the absence of a susceptible vertebrate host. In this study, designed to test whether Zika virus (ZIKV) could be vertically transmitted, female *Aedes aegypti* and *Aedes albopictus* were injected with ZIKV, and their F_1_ adult progeny were tested for ZIKV infection. Six of 69 *Ae. aegypti* pools, comprised of a total of 1,738 F_1_ adults, yielded ZIKV upon culture, giving a minimum filial infection rate of 1:290. In contrast, none of 803 F_1_ *Ae. albopictus* adults (32 pools) yielded ZIKV. The MFIR for *Ae. aegypti* was comparable to MFIRs reported for other flaviviruses in mosquitoes, including dengue, Japanese encephalitis, yellow fever, West Nile, and St. Louis encephalitis viruses. The results suggest that vertical transmission may provide a potential mechanism for the virus to survive during adverse conditions.

## Introduction

Zika virus (ZIKV) is a mosquito-transmitted flavivirus in the family *Flaviviridae*. During the past decade, ZIKV has moved from the status of an obscure arthropod-borne virus (arbovirus) of little public health importance to the position of a Public Health Emergency of International Concern (PHEIC).^1^–^3^ Apart from reports of sexual and congenital transmission in humans, the primary transmission cycle of ZIKV is thought to involve primates (nonhuman as well as human) and certain species of *Aedes* mosquitoes.^1^,^2^ The available data indicate that there are two cycles of ZIKV; a sylvan cycle involving nonhuman primates and forest-dwelling mosquitoes, and an urban/suburban cycle involving humans and *Aedes aegypti* and, to a lesser extent, *Aedes albopictus*.^1^,^2^ In this regard, the sylvan and urban cycles of ZIKV are similar to those described for dengue virus (DENV), yellow fever virus (YFV), and chikungunya virus, the three other arboviral pathogens that are also transmitted by *Ae. aegypti* in their urban cycles.^4^–^6^

Since the declaration of the current Zika epidemic as a PHEIC, ZIKV research has focused mainly on the pathogenesis, genetics, molecular biology, and structure of the virus, with the goal of developing improved diagnostic methods, vaccines, therapeutics, and effective methods of disease prevention.^3^ In contrast, much less research effort has been directed at the mosquito component of the ZIKV life cycle, specifically factors affecting vector competence, transmission efficiency, and long-term maintenance of the virus.

One of the basic questions that has long puzzled arbovirologists is how arboviruses persist during adverse environmental conditions (cold periods in temperate regions and hot dry seasons in tropical zones) when adult vectors, such as mosquitoes, are absent or in very low numbers.^7^ Vertical (transovarial or transovum) transmission (VT) of a virus from female insects directly to their progeny is one mechanism for arbovirus maintenance in nature during adverse environmental conditions. VT can also maintain a virus in a specific locality, when most of the potential vertebrate hosts are immune, either as a result of vaccination or natural infection. There is both field and laboratory evidence that many arboviruses are vertically transmitted in their natural arthropod vectors, including some of the major flaviviruses such as DENV, YFV, Japanese encephalitis virus (JEV), St. Louis encephalitis virus (SLEV), West Nile virus (WNV), and tick-borne encephalitis virus.^7^ There is also evidence that many of the insect-specific nonpathogenic flaviviruses are also maintained by VT in their natural mosquito hosts.^8^,^9^

Given this background, we undertook laboratory studies to determine if ZIKV is vertically transmitted in *Ae. aegypti* and *Ae. albopictus*. This report describes results of our preliminary studies, demonstrating that ZIKV is vertically transmitted by infected *Ae. aegypti* females to some of their progeny, thus providing a potential mechanism for the virus to be maintained during adverse climatic conditions.

## Materials and Methods

### Mosquitoes.

Two established laboratory colonies of *Ae. aegypti* and *Ae. albopictus* at the University of Texas Medical Branch were used in this study. The progenitors of the *Ae. aegypti* colony were originally obtained from Bangkok, Thailand; the progenitors of the *Ae. albopictus* colony were originally collected in Maracaibo, Venezuela. Both colonies were determined to be free of insect-specific viruses (ISVs) by culture in C6/36 cells, next-generation sequencing, and transmission electron microscopy, since previous studies^8^,^9^ have shown that some mosquito laboratory colonies are infected with ISVs that may reduce the infectivity and replication of a second heterologous flavivirus by superinfection exclusion. Mosquitoes were reared in an insectary, maintained at 27°C with 80% relative humidity and a 16-hour light/8-hour dark photoperiod.

### Virus.

The virus used in the experiments was ZIKV strain MEX I-44. This virus was originally isolated from a pool of *Ae. aegypti* mosquitoes collected in Chiapas, Mexico, in December 2015 and had been passaged three times in Vero cells.

### Infection of mosquitoes.

Approximately 100 female mosquitoes of each species were inoculated intrathoracically^10^ with a ZIKV stock containing 10^6^ plaque forming units (PFU)/mL. Infected mosquitoes were held in screened cages (BioQuip Products, Gardena, CA) within a plastic glove box at 27°C and maintained on 10% sucrose solution. Ten days after infection, mosquitoes were fed defibrinated sheep blood, using a Hemotek membrane feeding system (Discovery Workshops, Accrington, United Kingdom), as per manufacturer's instructions. After feeding, approximately 50 blood-engorged females were removed from the cage and transferred into four cylindrical 5-L cardboard containers with fine netting on top, each containing a 50-mL beaker holding moist paper toweling for oviposition.^10^ Cotton balls saturated with 10% sucrose solution were placed on top of the containers as an energy source. Six days after the blood meal, when many eggs were present on the moist paper, it was removed from the cage and dried for storage. Ten of the surviving female mosquitoes of both species were removed from the cages and frozen for subsequent titration.

### Virus assay on mosquitoes.

Eggs from the first oviposition of the infected *Ae. aegypti* and *Ae. albopictus* females were hatched and the emerging larvae reared to adults, using standard procedures^10^ in an insectary maintained at 27°C. The F_1_ adult offspring from the infected parents were collected and frozen at −80°C. Frozen F_1_ adult mosquitoes were subsequently thawed and sorted into pools of 25 insects (both sexes) each. Mosquito pools were then homogenized in 1.0 mL phosphate-buffered saline, pH 7.4, containing 20% fetal bovine serum with penicillin (100 U/mL) and streptomycin (100 mg), using a TissueLyser (Qiagen, Hilden, Germany). After centrifugation at 10,000 rpm for 10 minutes, the supernatant was passed through a 0.20-μm nylon syringe filter (Fisher Scientific, Pittsburgh, PA); then 150 μL of each supernatant filtrate was inoculated into separate 12.5-cm^2^ flask cultures of C6/36 cells, originally obtained from the American Type Culture collection (Manassas, VA). After 2 hours of absorption at 28°C, 5.0 mL of maintenance medium was added to each flask. Cultures were held in an incubator at 28°C for 7 days. On the seventh day, some of the cells were scraped from the plastic surface, and spotted onto Cel-Line 12-well glass slides (Thermo Fisher Scientific, Waltham, MA) for examination by indirect fluorescent antibody technique (IFAT),^11^ using a specific mouse hyperimmune polyclonal antibody prepared against ZIKV strain MR 766, obtained from the World Reference Center for Emerging Viruses and Arboviruses.

### RNA extraction and quantitative real-time reverse transcription polymerase chain reaction.

RNA extraction from the ZIKV-infected parent female mosquitoes was performed using a combination of TRIzol reagent (Life Technologies) and Qiagen protocols, as we have previously optimized the combination of these protocols to yield high-quality and high-integrity RNA.^12^ After extraction, all RNA samples were quantified spectrophotometrically using a NanoDrop ND-1000 (NanoDrop Technologies, Thermo Fisher Scientific). A real-time primer/probe set specific for ZIKV MR766 (Prototype, Uganda, 1947, GenBank accession no. AY632535)^13^ was synthesized by IDT Technologies (Coralville, IA) with 5′FAM as a reporter dye for the probe. Quantitative and reverse transcription polymerase chain reaction (RT-PCR) steps were performed with iScript One-Step RT-PCR Kit for Probes (Bio-Rad, Hercules, CA) using the manufacturer's protocol and an iCycler (Bio-Rad). For absolute quantification, a standard curve was constructed with 10-fold dilutions of RNA extracted from a sample of known infectivity of 2.03 × 10^6^ PFU determined by plaque assay in Vero E6 cells. Amplification efficiency of the reaction was 100.7% with a correlation coefficient of 0.99. A linear equation was generated by plotting the threshold cycle (*C*_T_) values of the standard curve and the log of the viral concentration. Viral load *C*_T_ values from the mosquito samples were determined by real-time PCR and converted to log_10_ PFU equivalents per mosquito, using the linear equation determined for the standard curve.^12^

## Results

### Virus load in parent females.

Ten of the parent female mosquitoes of each species (*Ae. aegypti* and *Ae. albopictus*) were frozen for testing 10 days after infection with ZIKV and 6 days after their first blood meal (total of 16 days postinfection). Each mosquito was tested individually by RT-PCR, as described above, and the viral load was calculated as log_10_ PFU per insect. The mean virus titer in the 10 *Ae. aegypti* females was 6.13 log_10_ PFU/mosquito (standard deviation [STDEV] = 0.57 log_10_). Mean virus titer in the 10 *Ae. albopictus* females was 6.35 log_10_ PFU/mosquito (STDEV = 0.45 log_10_).

### Testing F_1_ progeny for ZIKV infection.

**Figure 1.**
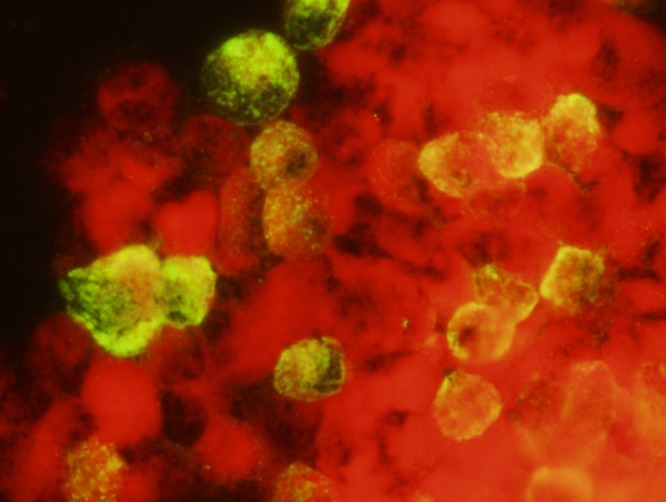
Zika virus antigen in C6/36 cells inoculated with a pool of 25 infected F_1_ *Aedes aegypti* adults, as detected by indirect immunofluorescent antibody technique.

A total of 69 pools consisting of 1,738 F_1_ adult *Ae. aegypti* were tested. Six of the 69 pools were IFAT-positive (Figure 1

), indicating that one or more of the F_1_ progeny in the pool were infected with ZIKV. Based on these results, we estimated a minimum ZIKV filial infection rate of 1:290 for the experimentally infected *Ae. aegypti*. None of the 32 *Ae. albopictus* pools, comprising 803 adults, were positive in the IFAT.

## Discussion

The observation that ZIKV was vertically transmitted by infected *Ae. aegypti* females to some of their F_1_ offspring is not unexpected, in view of the results of other studies of VT with flavivirus pathogens in their mosquito vectors.^14^–^27^ Table 1 summarizes the results of selected published studies of VT of nine flavivirus pathogens by mosquitoes. In these studies, the parent female mosquitoes were infected by intrathoracic inoculation; although this is not a natural route of infection, the technique insures that all of the females are infected. In contrast, infection of mosquitoes by the oral route requires high titers of virus and the results are more variable. However, even in experiments in which 100% of the parent females were infected, the filial infection rates in their F_1_ offspring varied widely, depending upon the mosquito species and geographic strain used, the virus type and strain used, the mosquito developmental stage tested (immature or adult), the virus assay method, the larval rearing temperature, the interval between initial infection and the first blood meal, and the ovarian cycle of F_1_ offspring examined.^14^–^27^ For example, several studies^16^,^17^,^20^,^22^,^25^ have compared the filial infection rates of DENV, JEV, or SLEV in multiple geographic strains of a single mosquito species (i.e., *Ae. aegypti*, *Ae. albopictus*, or *Culex quinquefasciatus*) and have reported marked differences in the infection rates of their F_1_ progeny. Similarly, other studies^16^,^22^–^24^ have compared the filial infection rates among different mosquito species after infection with a single flavivirus type. Alternatively, other studies^16^,^17^,^22^,^26^ have compared filial infection rates in a single mosquito colony experimentally infected with multiple strains of a single flavivirus type. In each case, considerable variation was observed in filial infection rates among F_1_ progeny of the infected female parents. Two studies also reported that lowering the larval rearing temperature to 18°C instead of 27°C significantly increased the filial infection rates of mosquitoes infected with SLEV.^16^,^23^–^25^ Two other studies^26^,^27^ examined the filial infection rates among progeny of individual infected female mosquitoes, and these also varied widely. In summary, experimental studies of VT of flaviviruses in mosquitos have given highly variable results, indicating that there are multiple factors (variables) that can affect the frequency of VT and the resulting filial infection rates in female mosquitoes infected with flaviviruses pathogens. If such variability occurs in laboratory studies that are performed under relatively controlled conditions, then it must be even greater in nature. Consequently, one should interpret the results of such laboratory studies with caution, as they simply demonstrate that VT of these flavivirus pathogens can occur. But the critical question is “does VT occur in nature”? Answering this question is a much more difficult problem, as one would have to collect immature forms (i.e., larvae and pupae) or adult males of the vector species in an area where the virus of interest is endemic or epidemic and to demonstrate that they are infected.

Another potential variable is the presence of ISVs. The earlier experimental studies mentioned above and in Table 1 were performed at a time when most of the mosquito-specific flaviviruses were still unrecognized,^8^,^10^ consequently the infection status of the mosquito colonies used, with regard to mosquito-specific viruses, was unknown. Available evidence now indicates that mosquito-specific flaviviruses are common in nature and in laboratory mosquito colonies.^8^,^10^ These viruses are also maintained by VT in their insect hosts and at much higher filial infection rates than the flavivirus vertebrate pathogens.^8^,^10^ The effect of these ISVs on the vector competence of their mosquito hosts is uncertain and may represent another important variable affecting VT of flavivirus arboviral pathogens, such as ZIKV, DENV, YFV, JEV, SLEV, and WNV.^8^,^10^ One potential effect of prior infection of a mosquito with an insect-specific flavivirus is superinfection exclusion (or homologous interference), a process by which cells infected with one virus do not support replication of the same or a similar virus.^9^ This phenomenon has been observed during infection of both vertebrate and invertebrate hosts with a broad range of viruses, including some of the mosquito-specific flaviviruses.^9^ Consequently, the absence or presence of mosquito-specific flaviviruses in a laboratory mosquito colony potentially could also alter the VT rate of a flavivirus pathogen in that colony.

In view of the relatively low filial infection rates observed with ZIKV and other flavivirus pathogens (Table 1), one might conclude that VT is of little epidemiologic importance. With the low filial infection rates observed in those experimental studies, mathematical models^28^,^29^ predict that flavivirus pathogens such as ZIKV, DENV, YFV, or WNV would survive for only a few generations without horizontal transmission and amplification in a vertebrate. However, VT rates in this range may be sufficient to allow a flavivirus to persist during hot dry periods or cold weather, when adult vectors are absent or in low numbers.^29^ Many *Aedes* species, including *Ae. aegypti* and *Ae. albopictus*, produce resistant eggs that can survive for months (or longer) in a dried dormant state.^30^,^31^ Although adults may not survive a winter or dry season, when favorable environmental conditions return, the eggs hatch and larvae emerge to complete the insect's life cycle and to establish a new generation. As the larvae pass through various developmental stages and grow, the virus also replicates, so that by the time an infected adult female emerges, she is infectious and able to transmit the virus.

Venereal transmission is another mechanism by which a virus can be amplified in a mosquito population. Although male mosquitoes do not take blood, they can acquire virus by VT from an infected female parent. In experimental studies, infected male mosquitoes can transmit virus horizontally to noninfected adult females during mating as well as to her developing oocytes, resulting in infected F_1_ progeny.^7^,^32^

Another mechanism for virus to be maintained in an insect population is the stabilized infection model, as described with Sigma virus (*Rhabdoviridae*) in *Drosophila melanogaster*.^33^ In this condition, the virus is maintained by VT at a high level in nature by relatively few infected females. Stabilized infections have been demonstrated in several *Aedes* mosquito species with California encephalitis serogroup viruses (*Orthobunyavirus*: *Bunyaviridae*).^34^,^35^

Because of these alternative methods of virus transmission, the importance of VT should not be discounted, based solely on low filial infection rates observed in experimental laboratory studies.

Knowledge of the transmission mechanisms of arboviruses in their vertebrate and invertebrate hosts continue to evolve. ZIKV was once thought to be transmitted to humans solely by the bite of infected mosquitoes. The recent pandemic in the South Pacific and the Americas has shown that other modes of human infection can occur (venereal, congenital, postpartum, and blood transfusion).^1^,^2^ The classical concept that arboviruses are maintained in nature by continual transmission between susceptible vertebrate hosts by hematophagous arthropods is also outdated and incomplete, as alternative mechanisms for virus maintenance within the vector population have been described. ZIKV is probably no exception. As a survival strategy, a successful parasite (in this case an arbovirus) might be expected to have alternative maintenance mechanisms to insure its survival during periods when susceptible vertebrate or arthropod hosts are not available. The alternative maintenance mechanisms (survival strategies) for ZIKV in its mosquito hosts is a neglected area of research, but this information is essential if we want to fully understand the ecology of ZIKV and to eventually control it.

## Figures and Tables

**Table 1:** Results of selected published studies of vertical transmission of flaviviruses by mosquitoes

Virus	Mosquito species	Filial infection rate(s)*	Reference
Yellow fever virus	*Aedes aegypti*	1:472 to 1:632	14
	*Haemagogus equinus*	1:5,245	15
	*Aedes mascarensis*	1:707	14
DENV-1	*Ae. aegypti*	< 1:600 to 1:1,543	16
	*Aedes albopictus*	1:200	16
	*Ae. albopictus*	1:217	17
DENV-2	*Ae. aegypti*	< 1:401 to < 1:459	16
	*Ae. aegypti*	1:813 to 1:3,042	18
	*Ae. albopictus*	1:408	16
DENV-3	*Ae. aegypti*	< 1:540	16
	*Ae. albopictus*	1:320	16
	*Ae. aegypti*	1:36	19
DENV-4	*Ae. aegypti*	< 1:1,700	16
	*Ae. albopictus*	1:194	16
West Nile (Kunjin) virus	*Culex tritaeniorhynchus*	1:325 to 1:850	20
	*Ae. aegypti*	1:62 to 1:72	20
	*Ae. albopictus*	1:471	17
Japanese encephalitis virus	*Ae. albopictus*	1:235 to 1:826	21
	*Aedes togoi*	1:83 to 1:173	21
	*Ae. albopictus*	1:267	17
	*Culex pipiens*	1:711	22
	*Culex quinquefasciatus*	1:1,336 to 1:6,400	22
St. Louis encephalitis virus	*Culex tarsalis*	< 1:140	23
	*Aedes taeniorhynchus*	1:181	24
	*Cx. quinquefasciatus*	1:1,120	24
	*Ae. albopictus*	1:494 to < 1:828	25
Zika virus	*Ae. aegypti*	1:290	Present study
	*Ae. albopictus*	< 1:803	

DENV = dengue virus.

* Reported filial infection rates vary depending on the virus strain and mosquito strain used.
